# LieRHRV system for remote lie detection using heart rate variability parameters

**DOI:** 10.1038/s41598-024-80480-5

**Published:** 2024-12-28

**Authors:** Moran Davoodi, Nitay Aspis, Yael Drori, Ido Weiser-Bitoun, Yael Yaniv

**Affiliations:** 1 Laboratory of Bioelectric and Bioenergetic Systems, Faculty of Biomedical Engineering, Technion-Israel Institute of Technology, Haifa, Israel; 2 Rappaport Faculty of Medicine, Technion-Israel Institute of Technology, Haifa, Israel; 3 Department of Internal Medicine “C”, Rambam Health Care Campus, 3109601 Haifa, Israel

**Keywords:** Camera, Machine learning, Remote polygraph, Electrophysiology, Software, Cardiology

## Abstract

The standard polygraph, or lie detector, is limited by its reliance on average heart rate, subjective examiner interpretation, and the need for direct subject contact. Remote photoplethysmography (rPPG) offers a promising contactless alternative, by using facial videos to extract heart rate variability (HRV). We introduce "LieRHRV," a remote lie detection algorithm based solely on extracted HRV parameters. To test the HRV parameter quality, we compared these parameters to HRV parameters extracted from ECG and photoplethysmography (PPG) records archived in five gold-standard ECG/PPG datasets. A prospective study of 39 healthy volunteers was also performed to evaluate the accuracy of lie detection based on PPG- or rPPG-derived HRV parameters. Effective HRV parameter extraction from both PPG and ECG sources was demonstrated, with comparable outcomes among 60% of the parameters on average with the publicly available datasets, and prospective study with 80% of the parameters. LieRHRV performance on ECG, PPG or rPPG (with parameters selected for PPG) exhibited an accuracy of 83.3 ± 3%, 87.3 ± 4% or 91.7 ± 3.5%, respectively. In comparison, the naïve model for ECG, PPG or rPPG data achieved an accuracy of 58.3 ± 3%, 61.0 ± 3% or 67.0 ± 5%, respectively. This study demonstrated the feasibility and effectiveness of LieRHRV, and offers a promising avenue for advancing lie detection technologies beyond polygraph limitations.

## Introduction

The polygraph, also commonly known as a lie detector, is a tool used for interrogating criminal suspects or screening candidates for positions that require confidentiality^1^. The individual being examined is attached to sensors that measure various physiological responses, such as breathing rate, heart rate, blood pressure and perspiration. While the reported accuracy rate was approximately 80%^2^, its widespread use is hindered by several factors. These include subjective interpretation of the collected signals, the potential for trained individuals to control their physiological responses, the need to physically connect the examinee to the measure tools and reliance on average physiological measures instead of their variability.

The current lie detector relies on average heart rate measurements. However, heart rate is influenced by dynamic and chaotic processes, and exhibits oscillations at varying frequencies over continuously shifting time scales^3^. Consequently, even during resting conditions, electrocardiogram (ECG) recordings in mammals display intricate beat-to-beat variations in heartbeat intervals^4^. Heart rate variability (HRV) has emerged as a significant physiological indicator, with changes in HRV closely correlating with stress levels. HRV parameters enable the distinction between physical strain and elevated sympathetic drive in response to stressful situations, such as medical emergencies^5^. Previous studies have demonstrated the potential of HRV for lie detection^6,7^. While lying, stress levels increase, which is associated with changes in heart rate variability due to heightened sympathetic activity. Changes in sympathetic activity affect short-range HRV parameters^8^. However, these studies relied on HRV parameters calculated on ECG signals which are not easy to connect.

To enhance the accuracy of the system and enable remote lie detection, additional physiological measurements have been proposed, including eye movement^9^, micro-expressions^10^, and facial expressions^11^. Nevertheless, while these measures do not necessitate direct contact with the subject, their individual accuracy as indices is not consistently high^1^. Photoplethysmography (PPG) is a more convenient and reliable method for heart rate detection^12^. Yet, extraction of HRV parameters from PPG recordings has not been fully characterized. Moreover, PPG-based watches or bands require direct connection to the subject, thereby overthrowing the advantages of remote lie detection. Remote photoplethysmography (rPPG) is a promising contactless technology that utilizes videos of faces to extract heart rate^13^. While high accuracy is achieved for average heart rate, the ability to extract HRV parameters was tested only for two linear HRV parameters^14^.

This work aimed to design a lie detection method termed “LieRHRV”, solely based on HRV parameters extracted from heartbeat intervals sensed by rPPG. To address all the above challenges, we first tested the ability to extract HRV parameters from gold-standard PPG data. We conducted prospective experiments to evaluate the quality of HRV parameters extracted from PPG compared to the gold-standard PPG data. We also explored the quality of HRV parameters extracted from rPPG compared to those extracted from PPG or ECG. Finally, we examined whether lies can be detected by “LieRHRV” using HRV parameters detected by rPPG, PPG, or ECG.

## Results

In this section, we conducted three types of comparisons. First, we compared HRV parameters extracted from ECG and PPG using publicly available datasets. The term “similar” refers to two groups of features being statistically equivalent within a margin defined by the effect size (see Statistical Analysis section). For this comparison, we used an effect size of 0.5 (for a given significance level, we identified the minimum effect sizes in each experiment that would be sufficient to determine equivalence), indicating that the difference between the group means did not exceed half of the pooled standard deviation. Second, we assessed the similarity of HRV extracted from video in a prospective experiment, using an effect size of 0.8. Finally, we demonstrated the feasibility of lie detection using a model based on HRV features that can be extracted from ECG, PPG, or video recordings (see Materials and Methods section).

### Comparison between HRV parameters extracted from PPG and ECG using publicly available datasets

**Fig. 1:**
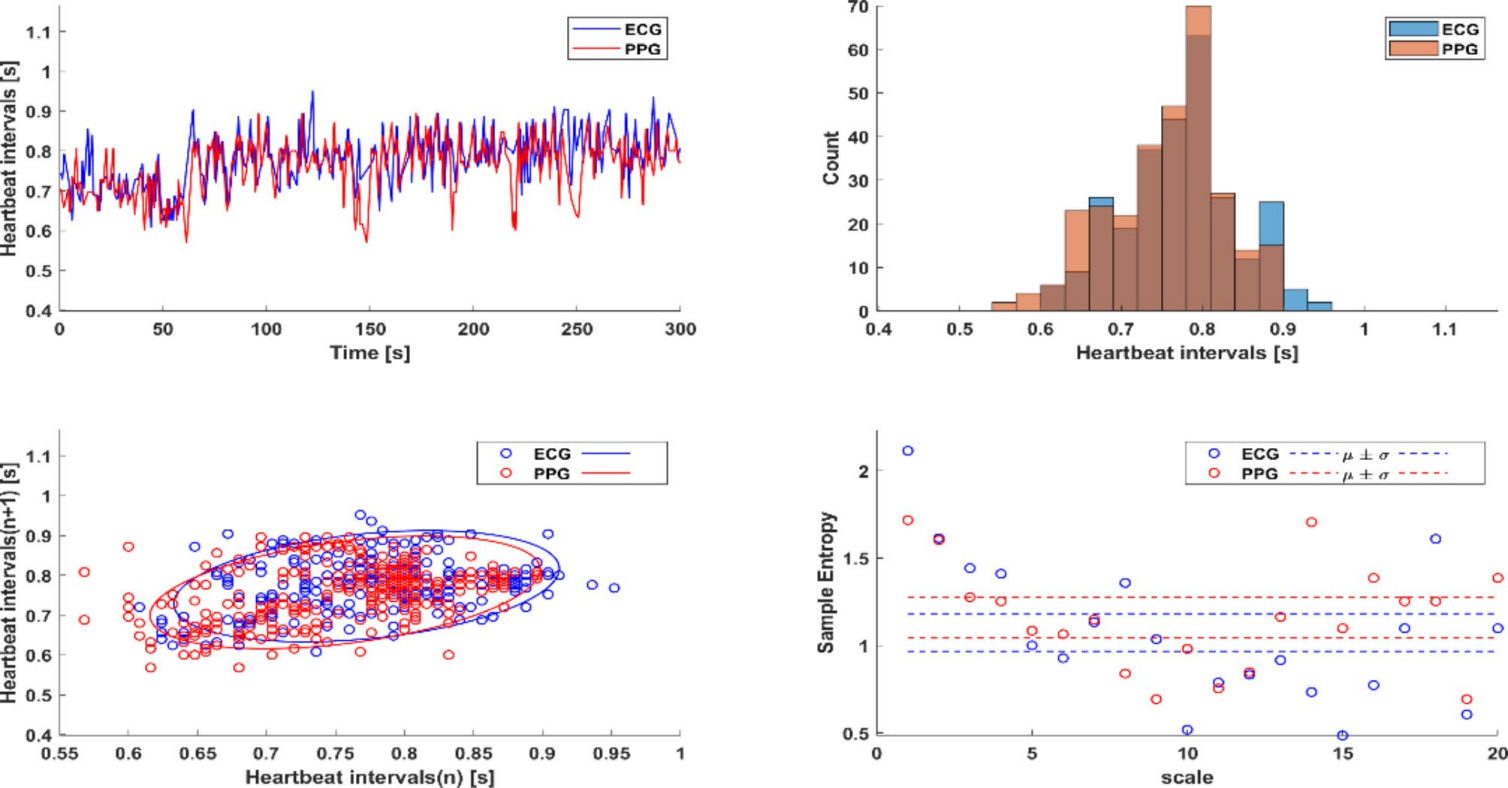
A representative example of heartbeat interval vs. time, heartbeat interval distribution, Poincaré plot and multi-scale entropy (MSE) extracted from ECG and photoplethysmography (PPG) records of patient #6 in the BIDMC dataset^15^.

**Fig. 2:**
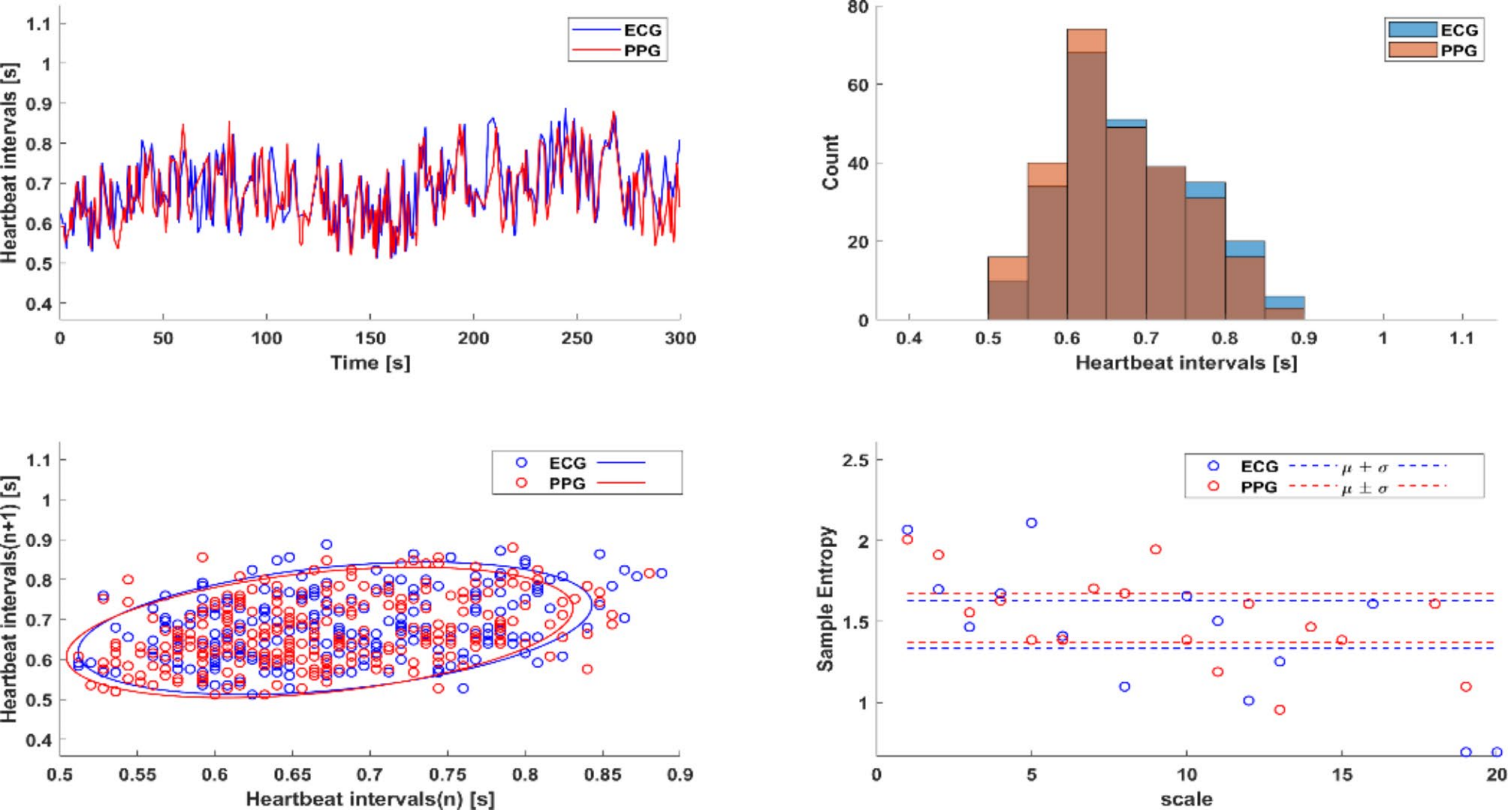
A representative example of RR interval vs. time, RR distribution, Poincaré plot and multi scale entropy (MSE) extracted from ECG and photoplethysmography (PPG) records of patient 5 in the MIMIC PERform AF dataset^16^.

To generate a standard for HRV parameters from PPG, we extracted HRV parameters from PPG and ECG records available in four different databases (refer to the Methods section). Figure 1 presents a representative example of heartbeat interval vs. time, heartbeat interval distribution, Poincaré plot and multiscale entropy (MSE) extracted from ECG and PPG. Table 1 provides a summary of statistics for all tested PPG-derived HRV parameters. Analysis of the BIDMC dataset^15^ found that 56.41% of the HRV parameters were similar when extracted from either PPG or ECG. The MIMIC PERform non-AF dataset^16^, which has recordings characterized by well spread atrial fibrillation arrhythmias, yielded similar results, albeit with a larger effect size due to the sample size. Figure 2 illustrates a representative example of heartbeat interval vs. time, RR distribution, Poincaré plot and MSE extracted from ECG and PPG for a patient with AF. Note that even under AF conditions, similar HRV parameters were extracted from PPG and ECG. In the majority of cases (64.1%), the PPG- and ECG-derived HRV parameters extracted from CapnBase^17^, which is considered the most reliable source of PPG^12^, were similar. Analysis of records in the WESAD^18^ database, which is based on a wristwatch and not pulse oximetry, resulted in 30.76% similarity between HRV parameters derived from PPG vs. ECG data.

**Table 1:** Summary of statistics for all tested heart rate variability parameters extracted from existing photoplethysmography (PPG) databases.

	BIDMC (N = 210)	CapnoBase (N = 158)	PERform_AF (N = 188)	PERform_non_AF (N = 150)	WESAD (N = 297)
AVNN	***	***	-	***	***
SDNN	***	***	-	***	-
RMSSD	***	***	-	***	-
SEM	***	***	-	***	-
PNN_50_	***	***	***	***	-
PIP	-	-	***	-	-
ILAS	-	-	***	-	-
PSS	-	-	***	-	-
PAS	-	-	***	-	-
HF NORM	-	-	***	-	-
HF PEAK	***	***	***	***	-
HF POWER	***	***	***	***	-
LF NORM	***	-	***	***	-
LF PEAK	***	***	***	***	***
LF POWER	***	***	***	***	-
LF/HF	***	-	***	-	-
VLF NORM	-	-	***	-	-
VLF POWER	-	-	***	***	-
TOTAL POWER	-	***	***	***	-
β	***	***	***	-	-
SD_1_	***	***	-	***	-
SD_2_	***	***	***	***	-
α_1_	-	-	***	***	-
α_2_	-	***	***	-	***
MSE1	-	***	***	-	***
MSE2	***	-	***	***	***
MSE3	***	***	***	***	***
MSE4	***	***	***	***	***
MSE5	***	***	***	***	**
MSE6	***	***	***	***	***
MSE7	***	***	***	***	***
MSE8	***	***	***	***	***
MSE9	***	***	***	***	***
MSE10	-	-	***	***	-
MSE11	-	-	***	-	-
MSE12	-	-	***	-	-
MSE13	-	***	***	-	-
MSE14	-	***	***	-	-
MSE15	-	***	***	-	-

– at least one of the two p values was above 0.05. *Both p values were below 0.05 and above 0.01. ** One p value was below 0.05 and above 0.01 and the other below 0.01. ***Both p values were below 0.01. Effect size = 0.5. N = number of windows. AVNN: average interval duration; SDNN: standard deviation of interval duration; RMSSD: the square root of the mean of the sum of the squares of differences between adjacent intervals; SEM: standard error; pNN50: percent of interval differences greater than 50 ms; PIP: percentage of inflection points in the heartbeat interval time series; ILAS: inverse average length of the acceleration/deceleration segments; PSS: percentage of short segments; PAS: the percentage of NN intervals in alternation segments; VLF: very low frequency; LF: low frequency; HF: high frequency; Norm: normalized; β: slope of the linear interpolation of the spectrum in a log–log scale for frequencies below the upper bound of the VLF band; SD_1_: interval standard deviation along the perpendicular to the line-of-identity; SD_2_: interval standard deviation along the line-of-identity; α_1_: DFA low-scale slope; α_2_: DFA high-scale slope; MSEx: multi-scale entropy scale x. Parameters in red are considered long-term heart rate variability indexes.

### Comparison between HRV parameters extracted from PPG and ECG using prospectively collected data

**Fig. 3:**
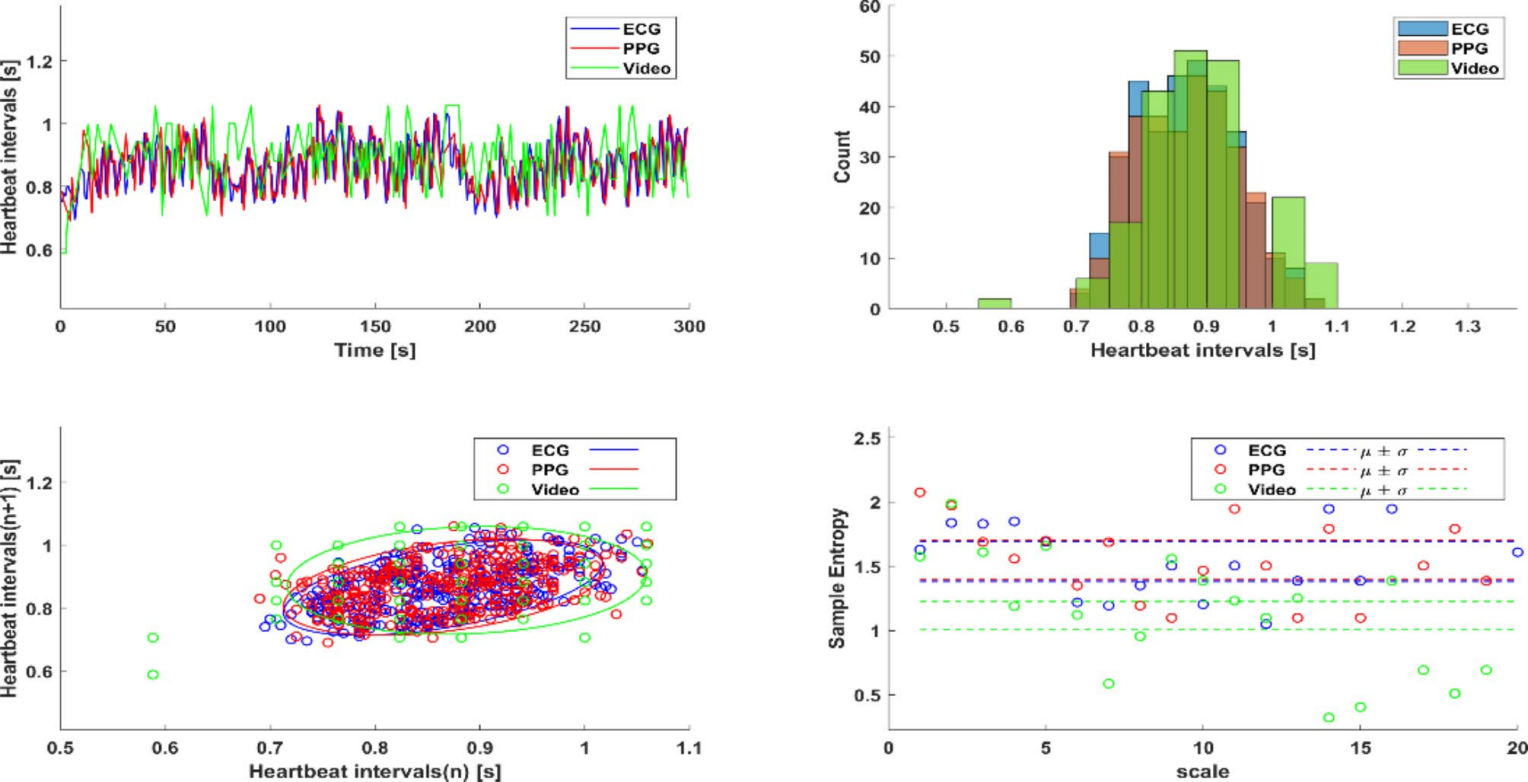
A representative example of heartbeat interval vs. time, heartbeat interval distribution, Poincaré plot and multi scale entropy (MSE) extracted from ECG, photoplethysmography (PPG) and remote PPG (rPPG) records of a volunteer in the perspective study.

To prospectively assess the similarity between HRV parameters extracted from PPG and ECG records, we collected ECG and PPG data from 39 healthy volunteers. Comparison revealed that 80% of the HRV parameters extracted from the two sets of data were similar. Figure 3 illustrates a representative example of heartbeat interval vs. time, heartbeat interval distribution, Poincaré plot, and MSE extracted from ECG and PPG data of a single volunteer. Table 2 summarizes the statistics for all tested HRV parameters.

**Table 2:** Summary of statistics for all tested heart rate variability parameters extracted from either ECG, photoplethysmography (PPG) or remote PPG (rPPG) records with an effect size of 0.8 from 39 volunteers.

	ECG vs. PPG (N = 483)	ECG vs. rPPG (N = 447)	PPG vs. rPPG (N = 447)
AVNN	***	***	***
SDNN	***	-	-
RMSSD	***	-	-
PNN_50_	-	-	-
SEM	***	-	-
PIP	-	-	-
ILAS	-	-	-
PSS	-	-	-
PAS	-	-	-
HF NORM	***	-	-
HF PEAK	***	***	***
HF POWER	***	-	-
LF NORM	***	-	-
LF PEAK	***	***	***
LF POWER	***	-	-
LF/HF	-	-	-
TOTAL POWER	***	***	***
VLF NORM	-	-	-
VLF POWER	***	***	***
β	***	-	-
SD_1_	***	-	-
SD_2_	***	-	-
α_1_	-	-	-
α2	***	-	-
MSE1	***	-	-
MSE2	***	***	***
MSE3	***	***	***
MSE4	***	***	***
MSE5	***	***	***
MSE6	***	***	***
MSE7	***	***	***
MSE8	***	***	***
MSE9	***	***	***
MSE10	***	-	-
MSE11	***	-	-
MSE12	***	-	-
MSE13	***	-	-
MSE14	***	-	-
MSE15	***	-	-

– at least one of the two p values was above 0.05. *Both p values were below 0.05 and above 0.01. ** One p value was below 0.05 and above 0.01 and the other below 0.01. ***Both p values were below 0.01. AVNN: Average interval duration; SDNN: Standard deviation of interval duration; RMSSD: The square root of the mean of the sum of the squares of differences between adjacent intervals; SEM: Standard error; pNN50: Percent of interval differences greater than 50ms; PIP: Percentage of inflection points in the heartbeat interval time series; ILAS: Inverse average length of the acceleration/deceleration segments; PSS: Percentage of short segments; PAS: The percentage of heartbeat intervals in alternation segments; VLF: very low frequency; LF: low frequency; HF: high frequency; Norm: normalized; β: Slope of the linear interpolation of the spectrum in a log–log scale for frequencies below the upper bound of the VLF band; SD_1_: interval standard deviation along the perpendicular to the line-of-identity; SD_2_: interval standard deviation along the line-of-identity; α_1_: DFA low-scale slope; α_2_: DFA high-scale slope; MSEx = Multi scale entropy scale x. Parameters in red are considered long-term heart rate variability indexes.

### Comparison between HRV parameters extracted from PPG and rPPG

To demonstrate the feasibility of remotely extracting HRV parameters, we compared parameters extracted from the ECG and PPG records collected from the 39 healthy volunteers to those extracted from rPPG records concurrently collected by face video. Figure 3 illustrates a representative example of heartbeat interval vs. time, heartbeat interval distribution, Poincaré plot, and MSE extracted from ECG, PPG, and rPPG of a single volunteer. Table 2 summarizes the statistics for all tested HRV parameters. Comparison of the results showed that 33.3% of the ECG- and rPPG-derived HRV parameters and that 33.3% of the PPG- and rPPG-derived HRV parameters were similar.

### Lie detection based on HRV parameters

To determine if lie detection and truth-telling can be distinguished using only HRV parameters, we separately evaluated our classification model with HRV data from three sources: ECG, PPG, and rPPG. For all systems, we focused on short-term HRV parameters. For ECG, we validated all short-term HRV parameters. We tested two approaches for using short-term HRV parameters from PPG data to differentiate between truth and deception. The first approach considered all short-term HRV parameters, while the second included only those statistically equivalent to ECG-derived HRV parameters. For rPPG data, we applied three approaches. Due to the limited number of participants with video quality suitable for lie detection, the first two approaches used the dominant short-term HRV parameters identified in either ECG or PPG data. In the third approach, we distinguished truth from lies using HRV parameters that were statistically equivalent to those from ECG or PPG.

**Fig. 4:**
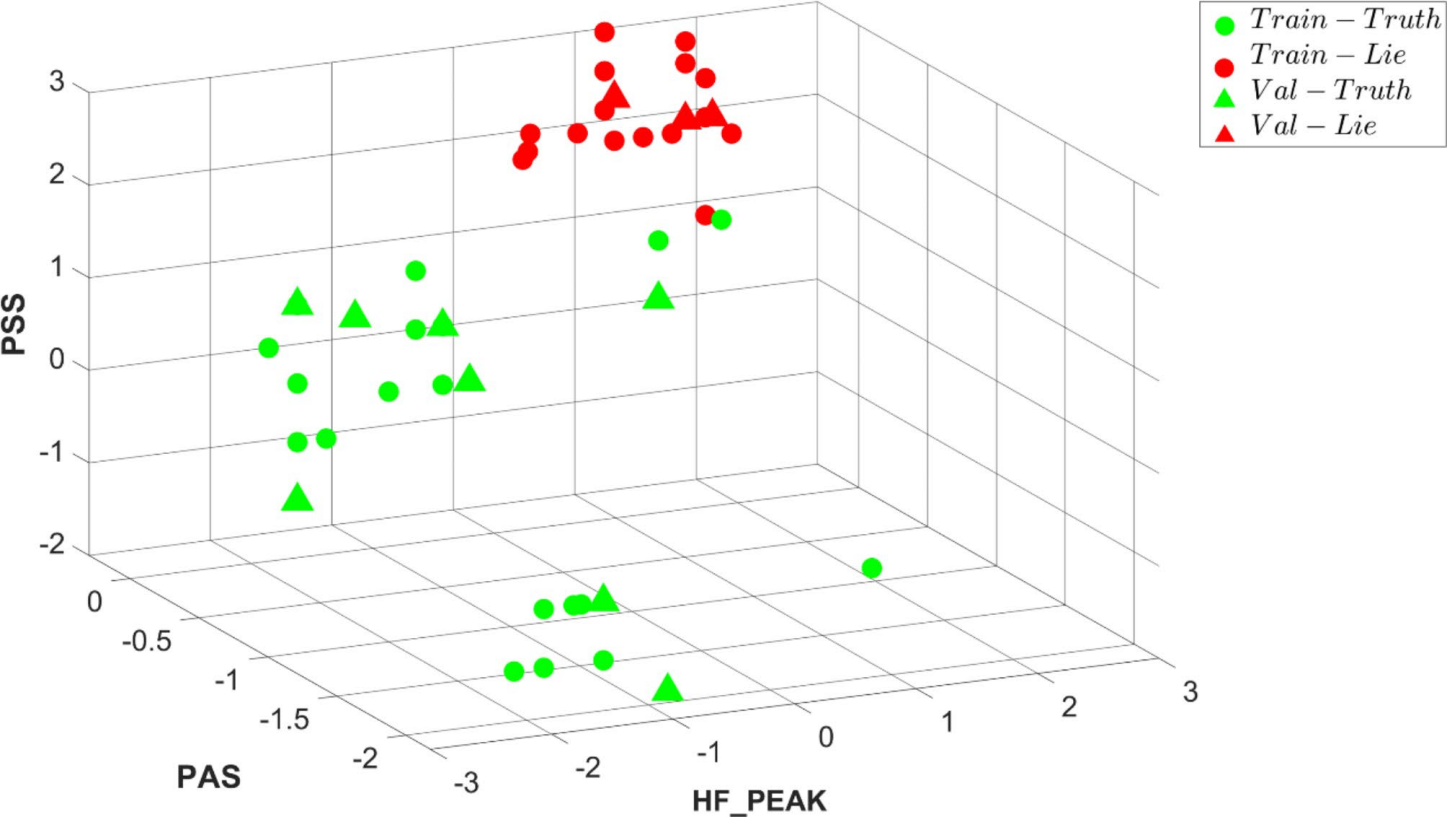
A representative example of lie and true detection from three heart rate variability parameters derived from an ECG record: high frequency (HF) peak, the percentage of intervals in alternation segments (PAS) and percentage of short segments (PSS). True-green, Lie-red, Circle-trained windows, *Triangle-*tested unseen windows.

When using ECG data, the dominant (see Methods for feature selection process) short-term HRV parameters distinguishing between true and lie were high frequency (HF) peak, the percentage of intervals in alternation segments (PAS) and percentage of short segments (PSS). A tenfold cross-validation was conducted for each patient, using an 80%-20% split for training and validation in each fold. In each evaluation for each patient we compare our model’s performances to a naïve one. We report the overall statistics of the times when our model performance was better or equal to the naïve one. Figure 4 illustrates a separation in 3D between true and lie segments based on ECG-derived HRV parameters. On average, our model was better at 93.3 ± 8% of the tests rather than a naïve model and had similar performance comparing to a naïve mode at 4.4 ± 6% of the tests (see definition in signal analysis under methods section). Our model accuracy was 83.3 ± 3% while the naïve model had an accuracy of 58.3 ± 3%.

**Fig. 5:**
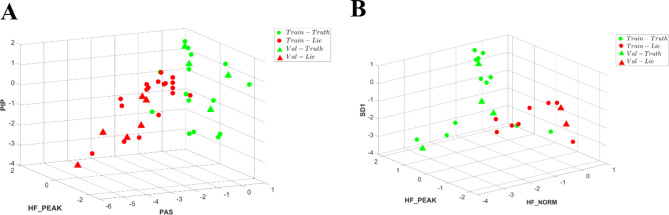
A representative example of lie and true detection from three heart rate variability parameters derived from a photoplethysmography (PPG) record: (**A**) high frequency (HF) peak, the percentage of intervals in alternation segments (PAS) and percentage of inflection points (PIP). (**B**) HF peak, HF norm and SD_1_. True-green, Lie-red, Circle-trained windows, *Triangle*-tested unseen windows.

We used two approaches to test if short-term HRV parameters from PPG data can be used to separate between true and lie. The first considered short-term HRV parameters and found that the dominant ones that separated between true and lie were HF peak, PAS and percentage of inflection points (PIP). Figure 5A illustrates a separation in 3D between true and lie segments based on PPG-derived HRV parameters. On average, our model was better at 90.0 ± 10% of the tests rather than a naïve model and had similar performance comparing to a naïve mode at 9.9 ± 10% of the tests. The accuracy of our model was 87.3 ± 4% and of the naïve was 61.0 ± 3%. In the second method, which used only the short-term HRV parameters that were statically equivalent to HRV parameters from ECG, HF peak, normalized HF and SD_1_ (heartbeat interval standard deviation of the first principal component of Poincaré Plot) were the dominant distinguishing features. Figure 5B illustrates a separation in 3D between true and lie segments based on PPG-derived short-term HRV parameters. On average, our model was better at 76.7 ± 23% of the tests rather than a naïve model and had similar performance comparing to a naïve mode at 19.9 ± 17% of the tests. The accuracy of our model was 87.8 ± 5% and of a naïve model was 66.0 ± 4%.

**Fig. 6:**
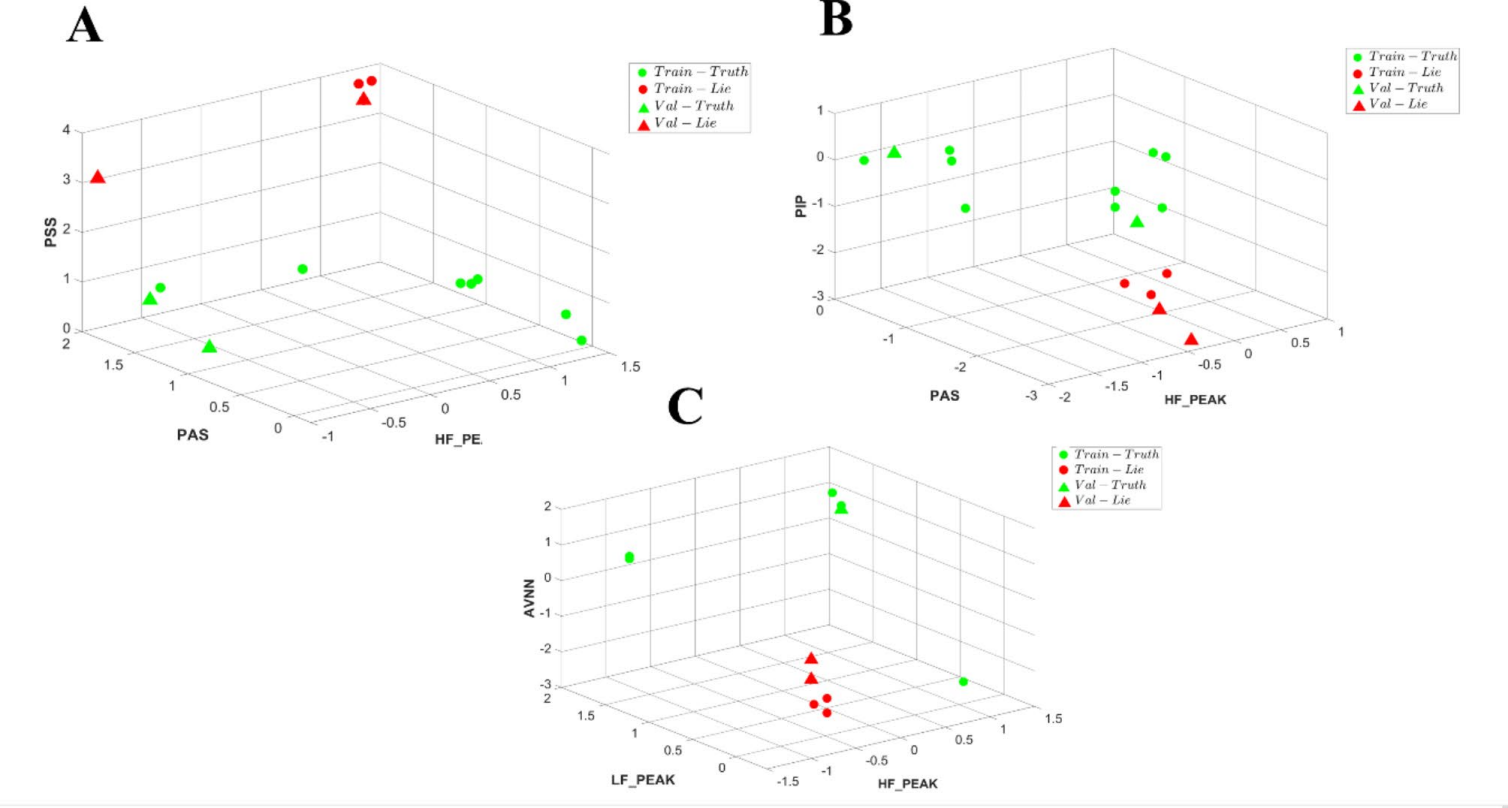
A representative example of lie and true detection from three heart rate variability parameters derived from a remote photoplethysmography PPG (rPPG) record: (**A**) high frequency (HF) peak, the percentage of intervals in alternation segments (PAS) and percentage of short segments (PSS). (**B**) HF peak, PAS and percentage of inflection points (PIP). (**C**) Average interval between normal heart beats (AVNN), HF peak, LF (low frequency) peak. True-green, Lie-red, Circle-trained windows, *Triangle*-tested unseen windows.

Three approaches were used to test if short-term HRV parameters from rPPG data can be used to distinguish between truth and lie. Figure 6A illustrates a 3D separation between true and lie segments based on ECG-derived short-term dominant HRV parameters. On average, “LieRHRV” outperformed the naïve model in 67.1 ± 18% tests and exhibited a similar performance of 28.6 ± 13%. “LieRHRV” achieved an accuracy of 89.8 ± 4%, whereas the naïve model achieved an accuracy of 69.0 ± 6%.

Figure 6B presents a 3D separation between true and lie segments based on the dominant short-term HRV parameters identified by PPG data. When employing the PPG-derived HRV parameters, our model surpassed the naïve model in 81.4 ± 15% tests and demonstrated a similar performance in 15.7 ± 14% tests. Our model achieved an accuracy of 91.7 ± 3.5%, while the accuracy of the naïve model was 67.0 ± 5%. Finally, we evaluated lie detection performance based on identified short-term HRV parameters that were statistically equivalent with effect size of 0.8 to short-term HRV parameters derived from either ECG or PPG records (average interval between normal heart beats (AVNN), HF peak, LF (low frequency) peak.

Figure 6C illustrates a 3D separation between true and lie segments based on identified short-term HRV parameters that were statistically equivalent with effect size of 0.8 to HRV parameters from either ECG or PPG. On average, “LieRHRV” outperformed the naïve model in 72.9 ± 12% tests and exhibited a similar performance of 20 ± 12% tests. “LieRHRV” achieved an accuracy of 87.3 ± 8%, whereas the naïve model achieved an accuracy of 66.5 ± 5%.

## Discussion

In this paper, we introduce a lie detection algorithm based on remotely detected HRV parameters that is also adaptable across various HRV-detection platforms. HRV parameters were extractable from both PPG and ECG sources, yielding comparable number of similar HRV parameters when tested on publicly available datasets or in a prospective study. Importantly, HRV parameters extracted from rPPG or from PPG or ECG achieved higher lie detection accuracy than the state-of-the-art lie detector^2^.

It has been previously demonstrated that heart rate can be extracted from PPG signals with a level of accuracy similar to that of ECG^12^. Previous research only compared a limited number of linear parameters^19^ or specific frequency parameters^20^. The current study was the first reported attempt to compare 39 different linear, frequency, and nonlinear HRV parameters derived from ECG, PPG or rPPG signals. Note that when HRV parameters are sensitive to changes in each beat interval (e.g., PIP, PAS, PSS) their comparability between PPG and ECG is poor. However, when statistical parameters (averaged over a certain number of beats) were compared, 94% were found to be equal when extracted from ECG vs. PPG sources. No significant relationship was found between time and frequency measurements when analyzing only short-term HRV.

Of note, even for patients with AF, the quality of HRV parameters derived from PPG signals was comparable to that of non-AF patients. Additionally, the analysis of the WESAD database revealed the lowest number of comparable HRV parameters between PPG and ECG signals. The limited number of samples available for analysis and the quality of the data in this database may have influenced the statistical outcomes.

The statistics from our comparative prospective study of HRV parameters extracted from ECG, PPG and rPPG records found PPG-derived parameters to be similar to those reported in published databases. Of note, when using either PPG or rPPG to extract HRV parameters, PIP, ILAS, PSS, PAS, and PNN50 were highly sensitive to beat-to-beat noise in the heart signal, and their comparison was poor. While high accuracy was achieved for average heart rate using rPPG^13^, the ability to extract HRV parameters was previously tested for only two linear HRV parameters^14^. Here, we tested all three types of HRV parameters. While for many HRV parameters the extraction performance was lower for rPPG than for PPG, the results were similar for sufficient parameters to detect lies.

We introduced a lie detection algorithm based on HRV parameters that can be implemented across various devices, including rPPG. While previous studies attempted to detect lies using HRV parameters^6,7^, our algorithm was the first to utilize PPG and rPPG, and not just ECG, as sources for HRV data. Despite the limited ability to extract HRV parameters from an rPPG source compared to ECG, the lie detection accuracy remained similar when we used only dominant short-term HRV parameters identified in either ECG and PPG data or identified short-term HRV parameters that were statistically equivalent with effect size of 0.8 to HRV parameters from either ECG or PPG. Similarly, the lie detection accuracy from PPG remained similar when we used only dominant short-term HRV parameters identified for PPG data or identified short-term HRV parameters that were statistically equivalent with effect size of 0.8 to HRV parameters from either ECG.

One of the limitations of the exist lie detector is the subjective examiner interpretation. Unlike subjective examiner interpretations, our lie detection algorithm performed automatic selection of lie and true criteria and therefore, does not depend on the experience of the examiner and can be performed automatically.

To detect lies, HRV parameters were employed, with a specific focus on short-term HRV parameters due to the assumed brief physiological response to lying. No significant change in performance was observed when using any of the sources (ECG, PPG, or rPPG). Thus, although the first signal comes from electrical activity and the latter two are derived from pulse waves, because the quantification of the beat interval is similar across all methods^12^, similar performance is obtained. When using an ECG source, the dominant HRV parameters for distinguishing between true and false statements were HF peak, PAS and PSS. Similarly, when using a PPG source, the dominant HRV parameters for differentiation were HF peak, PAS and PIP. When using an rPPG source, the dominant HRV parameters that were statistically equivalent with effect size of 0.8 to HRV parameters from either ECG or PPG used for differentiation were HF peak, LF peak and AVNN. The HF component is regulated in humans by the balance between the sympathetic and parasympathetic divisions of the automatic nervous systems^21^. Additionally, both PAS and PSS are influenced by internal pacemaker mechanisms^22^. Hence, signals from both neuronal and internal pacemaker systems enable accurate detection of lies.

### Applications

LieRHRV can have multiple applications. It can be used to interrogate highly dangerous subjects without their ability to interfere with the lie detection process. It can also be used for false statements in hospitals. For example, if a woman has been assaulted and there is a suspicion she is subjected to a threat to tell a lie.

### Limitations

The accuracy of the rPPG peak detection affects the quality of the extracted HRV parameters. Even though our lie detection algorithm achieved higher accuracy than state-of-the-art machines, improving the quality of beat interval series may further enhance the lie detection results.

We performed rPPG from a distance of 1 m, ensuring a sufficient region of interest to capture each patient’s face. Future experiments will have to validate if a longer distance can achieve similar results.

### Conclusions

In this paper, we demonstrate the feasibility of a remote lie detection system based on HRV parameters extracted from beat interval series of rPPG records. Such a system can function independently to detect lies or can be integrated with other physiological measures such as breathing rate, blood pressure and perspiration.

## Materials and methods

In this paper, we conducted several experiments. Initially, we compared HRV parameters extracted from PPG and ECG utilizing publicly available datasets. Subsequently, we carried out a prospective study to extract heartbeats intervals using ECG, PPG, and rPPG, and we compared their HRV parameter statistics. Finally, we presented a lie detection algorithm based on HRV that can be implemented across various devices. In this section, we introduce the ECG and PPG databases used, the experimental system, including participant statistics, the peak detection algorithm, HRV parameter calculation method, and the statistical method for comparison, as well as the lie detection algorithm.

### Publicly available databases

The **WESAD (Wearable Stress and Affect Detection) dataset**^18^ comprises data from 15 healthy subjects, and includes blood volume pulse, electrocardiogram, electrodermal activity, electromyogram, respiration, body temperature and three-axis acceleration. Measurements were conducted during rest and across three distinct affective states (meditation, stress, and amusement). The ECG was sampled at a frequency of 700 Hz, while the PPG was sampled at a frequency of 32 Hz.

The **CapnoBase** dataset^17^ contains ECG signals, PPG recordings obtained from pulse oximetry and capnography signals acquired from 42 patients during elective surgery and routine anesthesia. The ECG and PPG were sampled at a frequency of 300 Hz.

The **MIMIC PERform AF** dataset^16^ comprises data recorded from 35 critically-ill adults during routine clinical care using a bedside monitor. The data were extracted from the MIMIC-III Waveform Database^23^. Both the ECG and PPG were sampled at a frequency of 125 Hz.

The **BIDMC (Beth Israel Deaconess Medical Center)** dataset^15^ was extracted from the MIMIC-II resource^24^. It includes 8-min windows of PPG recordings, ECG recordings, and respiratory signals using conventional impedance pneumography acquired from 53 adults. The patients were randomly selected from a larger cohort admitted to ICUs for medical and/or surgical treatment. Both the ECG and PPG were sampled at a frequency of 125 Hz.

## Prospective experiments

### Participants

Healthy adult participants were recruited through public advertisement. The cohort included 16 men (mean age = 28 ± 3.4 years, mean BMI = 24.5 ± 3.4 kg/m^2^) and 23 women (mean age = 30 ± 7.3 years, mean BMI = 22 ± 3.8 kg/m^2^). The study adhered to the principles of the Declaration of Helsinki, and its protocol was approved by the Technion Institutional Review Board and Human Subjects Protection (140–2022). Informed consent from all patients was obtained.

### Recording system

ECG was recorded with a BIOPAC MP36 system (BIOPAC Systems, Inc., USA) in conjunction with hemoglobin saturation recorded through PPG, all with a sampling rate of 200 Hz. The ECG recordings were obtained using SS2LB electrodes (BIOPAC Systems, Inc., USA) and disposable F50 patches attached to both hand wrists and the left ankle. PPG was recorded using an SS4LA electrode (BIOPAC Systems, Inc., USA) connected to a PPG sensor^25^ operating in the infrared (IR) light range of 700–1000 nm. The diffuser plate allowed light entrance with a peak at 940 nm, and the photodetector had a peak at 870 nm. Additionally, two Lenovo tablets (TAB 2 A7_30H), each equipped with a 2MP video camera, recorded the participants at a sampling rate of 12–17 fps. One tablet, positioned approximately 1 m away from the participant, recorded the face, while the other, located about 30 cm from the participant, recorded the right hand. Synchronization of the cameras and the BIOPAC device recording was done manually.

### Experimental protocol

Participants were instructed to lie in a supine position on a hospital bed. For the baseline signal recording, participants rested for 15 min. Thereafter, they were asked six Yes/No questions over a 12-min period. When a continuous sympathetic stimulation is generated, a sustained elevation in heart rate is typically achieved within 20 to 30 s^26^. We should also notice that even a brief sympathetic stimulus (and not continuous) can affect HRV for 5 to 10 (sec). The response to vagal stimulation is nearly immediate, as can be observed with actions such as breathing, which is linked to the vagal nerve. Thus, participants were specifically instructed to wait for two seconds before responding and then to always answer “Yes.” On average, half of the “Yes” responses corresponded to true statements, while the other half were responses to false statements. Recording continued during the 2-min rest between each question.

## Signal analysis

Beat intervals were computed from all three signals. In the case of an ECG signal, the intervals were determined by the time difference between two consecutive R peaks. For a PPG signal, the intervals were determined by the time difference between two consecutive absorption peaks. In an rPPG signal, optical magnification was used to enhance subtle skin color variations caused by pulsatile blood flow. Color amplification and signal extraction were carried out using the “iPhys” application with the Independent Component Analysis (ICA) method^27^. The beat intervals were extracted as the peaks of the varying color signal.

**Fig. 7:**
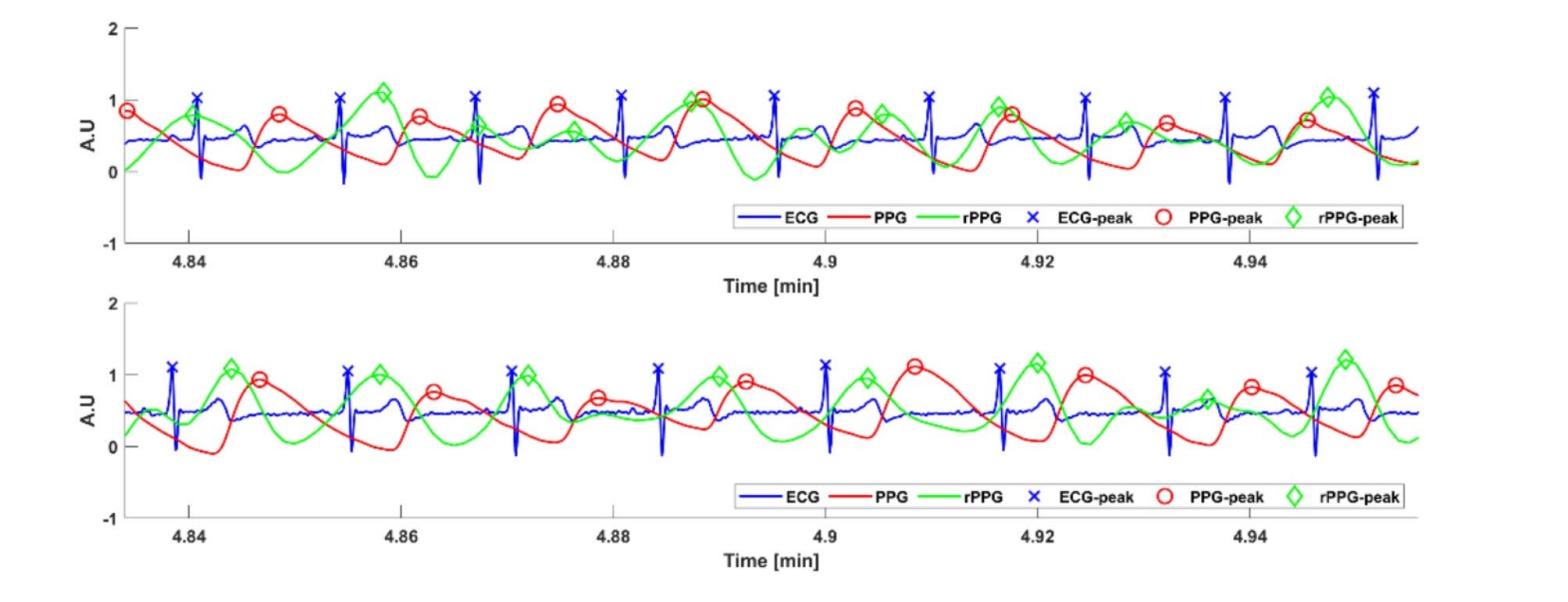
A representative example of ECG (in blue), photoplethysmography (PPG) (in dark red) and remote PPG (rPPG) (in dark green) signals from the prospective study. Detected peaks are shown with red x, green circle and blue diamond, respectively. Heartbeat interval intervals are the time intervals between peaks.

A distinct peak detection algorithm was necessary for each signal. ECG peak detection (R peaks) employed an agreement between the “jqrs” algorithm implemented in the PhysioZoo platform^28^ and the “rpeakdetect” algorithm^12^ (Fig. 7). PPG peak detection utilized the “qppg” algorithm^29^ for open databases and the “ampd” algorithm for prospective data^30^. Skin color variation peak detection was carried out using the “ampd” algorithm^30^. Once the peak locations were identified, they were converted into time intervals using the sampling frequency specific to each device.

The RR time series from each device were then used as the input for the PhysioZoo platform^28^ to extract short-term HRV parameters with a time window of 1 min and overlap of 30 s. For the long-term HRV parameters we used a 5 min window with no overlapping. The list of HRV parameters is provided in Behar et al.^28^.

### Lie detection algorithm

We define two periods:

1. Baseline, during which HRV parameters are calculated during 15 min of rest conditions.
2. Active state, during which HRV parameters are calculated for 1 min after a Yes/No question is asked.

**Fig. 8:**
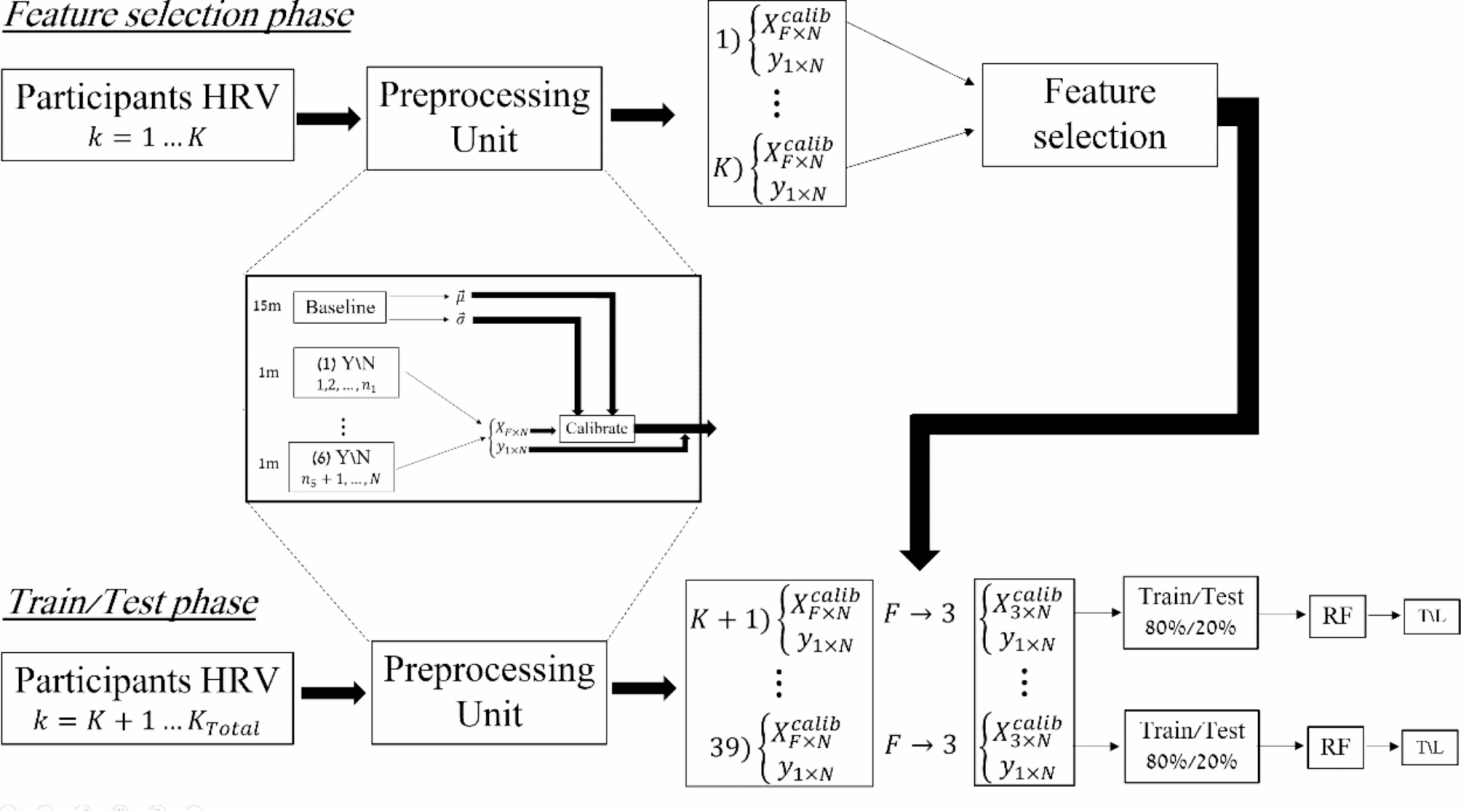
Schematic description of the lie detection algorithm. F is the number of heart rate variability (HRV) parameters, K_Total_ is the number of volunteers, σ is the average value of each HRV parameter, µ is the standard deviation of each heart rate (HR), ${x}_{fn}$ is each HRV parameter per window, ${y}_{n}$ is a binary label for a true or false statement, RF = random force, Y = Yes, N = No, T = true, L = lie.

Our main strategy was to compare the “active state” measure to baseline measures (see Fig. 8). When a continuous sympathetic stimulation is generated, a steady level of high heart rate reaches within 20–30 s. Even a brief sympathetic stimulus (and not continuous) can affect HRV for 5 to 10 s. The response to vagal stimulation is nearly immediate, as can be observed with actions such as breathing, which is linked to the vagal nerve^31^. Thus, to calculate HRV parameters during the baseline period, we used 30-s windows with two beats overlapping per patient. Subsequently, the next window began two beats forward with respect to the previous one. For example, if every interval is on average 1 s long, for a 15-min recording, we would end up with around 400 vectors.

The mean baseline HRV is noted as $$\left(\overrightarrow{\mu }=\left[\begin{array}{c}{\mu }_{1}\\ \vdots \\ {\mu }_{F}\end{array}\right]\right)$$, where F is the number of HRV parameters. The standard deviation vector, which is composed of the standard deviation of each HRV parameter, is noted as $$\left(\overrightarrow{\sigma }=\left[\begin{array}{c}{\sigma }_{1}\\ \vdots \\ {\sigma }_{F}\end{array}\right]\right)$$.

Similarly, in the active states, we used 30-s windows with two beats overlapping per patient per question. As mentioned in the introduction, when a continuous sympathetic stimulation is generated, a steady level of high HR reaches within 20–30 (sec). We should also notice that even a brief sympathetic stimulus (and not continuous) can affect HRV for 5 to 10 (sec). Since there was a two-minute break between questions and there was an overlapping of two beats for every window, it provided us at least five HRV windows labeled as telling a lie. Here, we calculated a measure (${x}_{fn}$) for each HRV parameter per window $\left({x}_{fn}, 1\le f\le F,1\le n\le N,\right)$, where n is a window index and N is the number of windows in all active states. Each feature was calibrated $\left({x}_{fn}^{calib}\right)$ to the baseline measure by:

1 $${\left\{{x}_{fn}^{calib}=\frac{{x}_{fn}-{\mu }_{f}}{{\sigma }_{f}},{y}_{n}\right\}}_{n=1}^{N}\forall f\in \left[1,F\right],$$

Where ${y}_{n}$ is a binary label for a true or false statement. Note that a higher ${x}_{fn}^{calib}$ represents greater change from baseline.

To select HRV parameters for training, we developed a scoring function aimed at detecting the maximum difference for each HRV parameters between false statements and true ones for a group of $P$ participants, referred to as the external group; training was executed with the selected HRV parameters on the remaining participants. For feature selection, we employed the following computations for every participant in the external group:

2 $${M}_{Lp}=\frac{1}{{N}_{Lp}}{\sum }_{{y}_{n}=0}{x}_{fn}^{calib}, {M}_{Tp}=\frac{1}{{N}_{Tp}}{\sum }_{{y}_{n}=1}{x}_{fn}^{calib}$$

Let ${N}_{Lp}$ and ${N}_{Tp}$ represent the values of single HRV sampled while telling a lie (${y}_{n}=0$) and the truth (${y}_{n}=1$), respectively, for patient $p$. We then separated participants based on whether their ${M}_{Lp}$ ​and ${M}_{Tp}$​ had the opposite (negative group) or the same (positive group) sign. For every patient in each group, we calculated the absolute value of the difference between ${M}_{Lp}$ and ${M}_{Tp}$​, and finally, computed the inverse of the coefficient of variation for each group. We denote the number of participants in the positive group as ${N}_{pos}$ ​and the number of participants in the negative group as ${N}_{neg}$​. The scoring function (*S*) for each feature was calculated as follows:

3 $$\begin{aligned}S= & \beta \frac{{\mu }_{pos}}{{\sigma }_{pos}}+\left(1-\beta \right)\frac{{\mu }_{neg}}{{\sigma }_{neg}}, \beta =0.45 \\ {\mu }_{pos}= & \frac{1}{{N}_{pos}}{\sum }_{p \in pos}\left|{M}_{Tp}-{M}_{Lp}\right|, {\sigma }_{pos}=\sqrt{\frac{1}{{N}_{pos}-1}{\sum }_{p \in pos}{\left(\left|{M}_{Tp}-{M}_{Lp}\right|-{\mu }_{pos}\right)}^{2}} \\ {\mu }_{neg}= & \frac{1}{{N}_{neg}}{\sum }_{p \in neg}\left|{M}_{Tp}-{M}_{Lp}\right|, {\sigma }_{neg}=\sqrt{\frac{1}{{N}_{neg}-1}{\sum }_{p \in neg}{\left(\left|{M}_{Tp}-{M}_{Lp}\right|-{\mu }_{neg}\right)}^{2}}\end{aligned}$$

The rationale behind this scoring function lies in its ability to measure separability between lies and truths on average across participants $\left({\mu }_{pos}, {\mu }_{neg}\right),$ while determining reliability by the standard deviations $\left({\sigma }_{pos}, {\sigma }_{neg}\right)$. The participants were separated into positive and negative groups to enable heavier weighting of the negative group, under the assumption that opposite signs of the means encapsulate more information for the classifying task, thereby exerting greater influence during the feature selection phase. In other words, assuming that a higher score from the negative groups contributes more to the classification task, an appropriate score would be a weighted average of both the positive and negative groups. Once all features are scored, we sort them in descending order of their scores and select the three features with the highest scores.

In the training phase, we utilized the data of participants who were not involved in the feature selection phase. We extracted only the three features identified in the feature selection phase and then constructed a set of training examples for each patient as described in Eq. (1), with the distinction that ${x}_{i}$ ​ is now a three-feature vector with the appropriate $\left(\mu ,\sigma \right)$ for each feature. Each patient’s training set was divided into training and validation sets with an 80%-20% ratio, respectively. A random forest model was applied to each participant’s training set. The models for different participants operated independently. Accuracy metrics were reported on the validation set and compared to those of a naïve classifier. The naïve classifier simply returns either 1 or 0 (truth or lie) per patient, depending on the majority label in the validation set; if there are more labels with the value of 1 in the validation set, it returns only 1; otherwise, it returns 0.

### Statistical analysis

When we have two groups that are assumed to be equivalent, we expect their mean difference to be approximately 0. The mean difference (which is a random variable with distribution) lies within some margin which is small enough to determine whether the difference is neglectable or not. In contrast to statistical significance, which tests whether there is any effect (or difference) at all, statistical equivalence focuses on whether an effect is sufficiently small to be considered negligible or practically equivalent to no effect. The practice of non-significant statistics $\left({p}_{value}>\alpha \right)$ as a way of determining equivalence is problematic because it is not possible to conclude that there is no effect when ${p}_{value}>\alpha$ which ignores the statistical power to detect a true effect. This is where effect size, like Cohen’s d, becomes important. One way to assess whether the effect is neglectable is to compare it to some standardized magnitude such as the ratio between the difference of mean values and some joint standard deviation. In the two one sided T-test (TOST)^13^ procedure, an upper $\Delta$ (${\Delta }_{U}$) and lower (${\Delta }_{L}$) equivalence bound is specified based on the smallest effect size of interest, for instance, 0.3 in Cohen’s d terms which means that the difference between the two groups means is no larger than 0.3 times the pooled standard deviation. Two composite null hypotheses are tested: H01: $\Delta <-$ ${\Delta }_{L}$ and H02: $\Delta >$ ${\Delta }_{U}$. Once the two are rejected, we state that groups are equivalent up to the boundaries of the specific margin. To determine whether the difference between all paired samples was neglectable, we used the one-sample version of TOST.

## Data Availability

All data are available upon request. Yael Yaniv yaely@bm.technion.ac.il.

## References

[1] Palena, N. & Caso, L. Investigative interviewing research: Ideas and methodological suggestions for new research perspectives. *Front. Psychol.* **12**, 715028 (2021). 34335429 10.3389/fpsyg.2021.715028 PMC8320696

[2] O’Sullivan, M., Frank, M. G., Hurley, C. M. & Tiwana, J. Police lie detection accuracy: The effect of lie scenario. *Law Hum. Behav.* **33**, 530–538 (2009). 19242785 10.1007/s10979-008-9166-4

[3] Goldberger, A. L., Rigney, D. R. & West, B. J. Chaos and fractals in human physiology. *Sci. Am.* **262**, 42–49 (1990). 2296715 10.1038/scientificamerican0290-42

[4] Billman, G. E., Huikuri, H. V., Sacha, J. & Trimmel, K. An introduction to heart rate variability: Methodological considerations and clinical applications. *Front. Physiol.* **6**, 55 (2015). 25762937 10.3389/fphys.2015.00055 PMC4340167

[5] Peabody, J. E., Ryznar, R., Ziesmann, M. T. & Gillman, L. A systematic review of heart rate variability as a measure of stress in medical professionals. *Cureus* **15**, e34345 (2023). 36865953 10.7759/cureus.34345 PMC9974008

[6] Swee, T. T. et al. Formulation of a novel HRV classification model as a surrogate fraudulence detection schema. *Malaysian J. Fundam. Appl. Sci.* **16**, 121–127 (2020).

[7] Paige, L. E., Wolf, J. M. & Gutchess, A. Evaluating heart rate variability as a predictor of the influence of lying on memory. *Memory* **30**, 785–795 (2022). 33258409 10.1080/09658211.2020.1849307

[8] Rosenberg, A. A., Weiser-Bitoun, I., Billman, G. E. & Yaniv, Y. Signatures of the autonomic nervous system and the heart’s pacemaker cells in canine electrocardiograms and their applications to humans. *Sci. Rep.* **10**, 1–15 (2020). 32561798 10.1038/s41598-020-66709-z PMC7305326

[9] Ge, F. F. et al. Application of eye tracker in lie detection. *Fa Yi Xue Za Zhi* **36**, 229–232 (2020). 32530172 10.12116/j.issn.1004-5619.2020.02.015

[10] Ben, X. et al. Video-based facial micro-expression analysis: A survey of datasets, features and algorithms. *IEEE Trans. Pattern Anal. Mach. Intell.* **44**, 5826–5846 (2022). 33739920 10.1109/TPAMI.2021.3067464

[11] Samuel, S., Chatterjee, T., Thapliyal, H. & Kacker, P. Facial psychophysiology in forensic investigation: A novel idea for deception detection. *J. Forensic Dent. Sci.* **11**, 90 (2019). 32082044 10.4103/jfo.jfds_49_19 PMC7006300

[12] Charlton, P. H. *et al.* Detecting beats in the photoplethysmogram: benchmarking open-source algorithms. *Physiol. Meas.* **43** (2022). 10.1088/1361-6579/ac826d PMC9393905 35853440

[13] Haugg, F., Elgendi, M. & Menon, C. GRGB rPPG: An efficient low-complexity remote photoplethysmography-based algorithm for heart rate estimation. *Bioeng. (Basel, Switzerland)* **10** (2023). 10.3390/bioengineering10020243 PMC9952130 36829737

[14] Pai, A., Veeraraghavan, A. & Sabharwal, A. HRVCam: Robust camera-based measurement of heart rate variability. *J. Biomed. Opt.* **26**, 22707 (2021). 10.1117/1.JBO.26.2.022707 PMC7874852 33569935

[15] Pimentel, M. A. F. et al. Toward a robust estimation of respiratory rate from pulse oximeters. *IEEE Trans. Biomed. Eng.* **64**, 1914–1923 (2017). 27875128 10.1109/TBME.2016.2613124 PMC6051482

[16] Bashar, S. K., Ding, E., Walkey, A. J., McManus, D. D. & Chon, K. H. Noise detection in electrocardiogram signals for intensive care unit patients. *IEEE Access Pract. Innov. Open Solut.* **7**, 88357–88368 (2019). 10.1109/access.2019.2926199 PMC7597656 33133877

[17] Karlen, W. CapnoBase IEEE TBME Respiratory Rate Benchmark. at 10.5683/SP2/NLB8IT (2021).

[18] Schmidt, P., Reiss, A., Duerichen, R., Marberger, C. & Van Laerhoven, K. Introducing WESAD, a multimodal dataset for wearable stress and affect detection. in *Proceedings of the 20th ACM International Conference on Multimodal Interaction* 400–408 (Association for Computing Machinery, New York, NY, USA, 2018). 10.1145/3242969.3242985.

[19] Janković, D. & Stojanović, R. Flexible system for HRV analysis using PPG signal BT - CMBEBIH 2017. in (ed. Badnjevic, A.) 705–712 (Springer Singapore, Singapore, 2017).

[20] Aygun, A., Ghasemzadeh, H. & Jafari, R. Robust interbeat interval and heart rate variability estimation method from various morphological features using wearable sensors. *IEEE J. Biomed. Heal. Inform.* **24**, 2238–2250 (2020). 10.1109/JBHI.2019.2962627 PMC11036325 31899444

[21] Weiser-Bitoun, I. et al. Age-dependent contribution of intrinsic mechanisms to sinoatrial node function in humans. *Sci. Rep.* **13**, 18875 (2023). 37914708 10.1038/s41598-023-45101-7 PMC10620402

[22] Shemla, O., Tsutsui, K., Behar, J. A. & Yaniv, Y. Beating rate variability of isolated mammal sinoatrial node tissue: Insight into its contribution to heart rate variability. *Front. Neurosci.* **14** (2021). 10.3389/fnins.2020.614141 PMC7928380 33679288

[23] Johnson, A. E. W. et al. MIMIC-III, a freely accessible critical care database. *Sci. Data* **3**, 160035 (2016). 27219127 10.1038/sdata.2016.35 PMC4878278

[24] Lee, J. et al. Open-access MIMIC-II database for intensive care research. *Annu. Int. Conf. IEEE Eng. Med. Biol. Soc. IEEE Eng. Med. Biol. Soc. Annu. Int. Conf.* **2011**, 8315–8318 (2011). 10.1109/IEMBS.2011.6092050 PMC6339457 22256274

[25] Chigira, H., Maeda, A. & Kobayashi, M. Area-based photo-plethysmographic sensing method for the surfaces of handheld devices. in *Proceedings of the 24th Annual ACM Symposium on User Interface Software and Technology* 499–508 (Association for Computing Machinery, New York, NY, USA, 2011). 10.1145/2047196.2047262.

[26] Victor, R. G., Seals, D. R. & Mark, A. L. Differential control of heart rate and sympathetic nerve activity during dynamic exercise. Insight from intraneural recordings in humans. *J. Clin. Invest.* **79**, 508–516 (1987). 3805279 10.1172/JCI112841 PMC424115

[27] McDuff, D. & Blackford, E. iPhys: An open non-contact imaging-based physiological measurement toolbox. *Annu. Int. Conf. IEEE Eng. Med. Biol. Soc. IEEE Eng. Med. Biol. Soc. Annu. Int. Conf.* **2019**, 6521–6524 (2019). 10.1109/EMBC.2019.8857012 31947335

[28] Behar, J. A. et al. PhysioZoo: A novel open access platform for heart rate variability analysis of mammalian electrocardiographic data. *Front. Physiol.* **9**, 1390 (2018). 30337883 10.3389/fphys.2018.01390 PMC6180147

[29] Vest, A. N. et al. An open source benchmarked toolbox for cardiovascular waveform and interval analysis. *Physiol. Meas.* **39**, 105004 (2018). 30199376 10.1088/1361-6579/aae021 PMC6442742

[30] Shin, H. S., Lee, C. & Lee, M. Adaptive threshold method for the peak detection of photoplethysmographic waveform. *Comput. Biol. Med.* **39**, 1145–1152 (2009). 19883905 10.1016/j.compbiomed.2009.10.006

[31] Bogdanov, K. Y. et al. Membrane potential fluctuations resulting from submembrane Ca2+ releases in rabbit sinoatrial nodal cells impart an exponential phase to the late diastolic depolarization that controls their chronotropic state. *Circ. Res.* **99**, 979–987 (2006). 17008599 10.1161/01.RES.0000247933.66532.0b

